# Nano Carbon‐mesh with Excellent Bonding Performance via Hydro‐cage De‐shielding Strategy

**DOI:** 10.1002/advs.75630

**Published:** 2026-05-13

**Authors:** Weijia Yang, Jianyong Wan, Hongda Guo, Haizhu Wu, Yongli Yang, Wenhe Bi, Zhengyong Yang, Chenghua Wang, Bertrand Charrier, Hisham Essawy, Antonio Pizzi, Xinyi Chen, Zhijun Chen, Guanben Du, Xiaojian Zhou

**Affiliations:** ^1^ Yunnan Provincial Key Laboratory of Wood and Bamboo Biomass Materials Southwest Forestry University Kunming China; ^2^ CNRS/Univ Pau and Pays Adour Institut des Sciences Analytiques et de Physico‐Chimie pour Environnement et les Matériaux‐Xylomat Mont‐de‐Marsan France; ^3^ Department of Polymers and Pigments National Research Centre Cairo Egypt; ^4^ LERMAB University of Lorraine Epinal France; ^5^ Key Laboratory of Bio‐based Material Science and Technology Northeast Forestry University Ministry of Education Harbin China

**Keywords:** nano carbon‐mesh, hydro‐cage De‐shielding, biomimetic structure, bio‐based adhesives

## Abstract

Carbon nanostructures (e.g., graphene, CNTs, MXenes) offer high strength and conductivity but suffer from severe agglomeration, instability, and complex synthesis, which hinder their scalable assembly into robust carbon networks. Meanwhile, the current methods for constructing nano carbon‐mesh (NCM) show enormous limitations, e.g., complex processes and enormous energy consumption, etc., making it difficult to achieve green, low‐carbon, and large‐scale applications. Here, an efficient NCM preparation method of hydro‐cage de‐shielding strategy to implement instantaneous carbonization‐polymerization was conceptualized. During the initial stage of hot‐pressing, cellulose‐based film (CF) carbonized into quasi‐spherical carbonized polymer dots (CPDs), where the system is containing enormous water, existed hydro‐cage shielding effect, which formed a huge barrier between CPDs, preventing CPDs from further polymerization. As the hot‐pressing progresses, water continuously evaporated or transformed from the free water state to the bound water state, the hydro‐cage shielding effect gradually weakened, the system formed a core of CPDs and further underwent polymerization and growth through its surface functional groups, gradually forming NCM similar to a dragonfly's wing with excellent toughness and load‐bearing capacity. As a result, the final NCM‐plywood achieved a wet shear strength of 1.24 ± 0.05 MPa under 63°C, exceeding the Class II plywood requirement (≥ 0.7 MPa).

## Graphical abstract

A hydro‐cage de‐shielding strategy transforms cellulose‐based films into a dragonfly‐wing‐like nano carbon‐mesh (NCM) adhesive through instantaneous carbonization‐polymerization. The resulting NCM‐plywood achieves exceptional wet shear strength (1.24 MPa at 63°C), exceeding Class II plywood standards, and retains 0.73 MPa after boiling‐water cycles. This green, scalable method offers a sustainable pathway for next‐generation high‐performance adhesives.

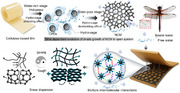

## 1 Introduction

Carbon nanostructures and porous frameworks such as graphene, carbon nanotubes (CNTs), MXenes, g‐C_3_N_4_, metal–organic frameworks (MOFs), and covalent organic frameworks (COFs), have enabled advances in energy storage, catalysis, separation membranes, and mechanical reinforcement owing to their high mechanical strength, conductivity, and tunable chemistry [1, 2, 3, 4, 5]. However, their strong agglomeration tendencies, oxidation or chemical instability, and reliance on complex coordination chemistry or multistep syntheses hinder their scalable assembly into continuous and robust carbon networks [6, 7]. These limitations highlight the need for a simple, energy‐efficient, and structurally deterministic approach to construct carbon architectures beyond conventional allotropes. Carbon dots (CDs), particularly carbonized polymer dots (CPDs), offer such potential due to their nanoscale dimensions, abundant surface functionalities, and hybrid core–shell structures [8, 9, 10, 11]. CPDs have been shown to form nano carbon‐mesh (NCM) through hydrophobic collapse–driven vesicular growth [12], topology‐retaining carbonization of hydrogen‐bonded or π–π assembled oligomers [13], hydrothermal dehydration–condensation–carbonization yielding “CPDs–polymer” interpenetrating networks [14], and hydrogel‐template routes producing three‐dimensional carbon skeletons [15, 16]. Yet, these routes still depend on hydrothermal or high‐temperature processing, complex precursors or templates and long reaction times, frequently resulting in poor morphological control, aggregation, broad functional‐group distributions, and limited reproducibility [12, 14, 16]. Thus, a green, rapid, and deterministic strategy for transforming polymeric precursors into continuous NCM remains critically needed. Such an advanced carbon architecture holds particular promise for developing next‐generation adhesives, as its continuous network and tunable surface chemistry could fundamentally address long‐standing issues of strength, toughness, and multi‐functionality in bonding layers [17, 18].

Conventional petroleum‐based adhesives, such as urea–formaldehyde and phenolic resins, suffer from intrinsic brittleness, poor water resistance, and formaldehyde emission, which are fundamentally incompatible with the goals of green and sustainable development [19, 20]. Furthermore, standard epoxy resin curing agents, such as diethylenetriamine and other petrochemical‐derived amines, raise significant environmental and health concerns due to the release of toxic volatiles during curing and the persistence of unreacted hazardous monomers within the resin matrix. This has driven increasing interest in bio‐based alternatives, such as nucleophilic amino acids (e.g., lysine and glutamic acid), which contain both amino and carboxyl groups capable of participating in epoxy ring‐opening reactions to form crosslinked networks, thereby offering a renewable and less toxic pathway for adhesive formulation [21]. Bio‐based adhesives present great environmental advantage, however, their bonding strength, durability, and functional versatility often remain insufficient for high‐performance applications [22, 23]. To overcome these limitations, incorporating nanomaterials such as carbon nanotubes, graphene, and nanocellulose into adhesive systems to construct nanocomposites has been widely recognized as an effective strategy [24, 25]. Nevertheless, most existing approaches face poor dispersion uniformity, as nanoparticles readily agglomerate due to strong interfacial interactions, generating defect sites within the adhesive layer [26]. In addition, limited chemical compatibility between externally added nanomaterials and organic matrices often leads to inefficient stress transfer [27]. Furthermore, nanoparticle pretreatment, functionalization, and dispersion introduce high process complexity and cost, thereby compromising the green nature of bio‐based systems [28, 29]. Consequently, developing a disruptive strategy that enables nanoscale reinforcing phases to be generated in situ, uniformly distributed, and chemically bonded within the matrix is critical for realizing next‐generation high‐performance and multifunctional adhesives.

## 2 Results and Discussion

**SCHEME 1:**
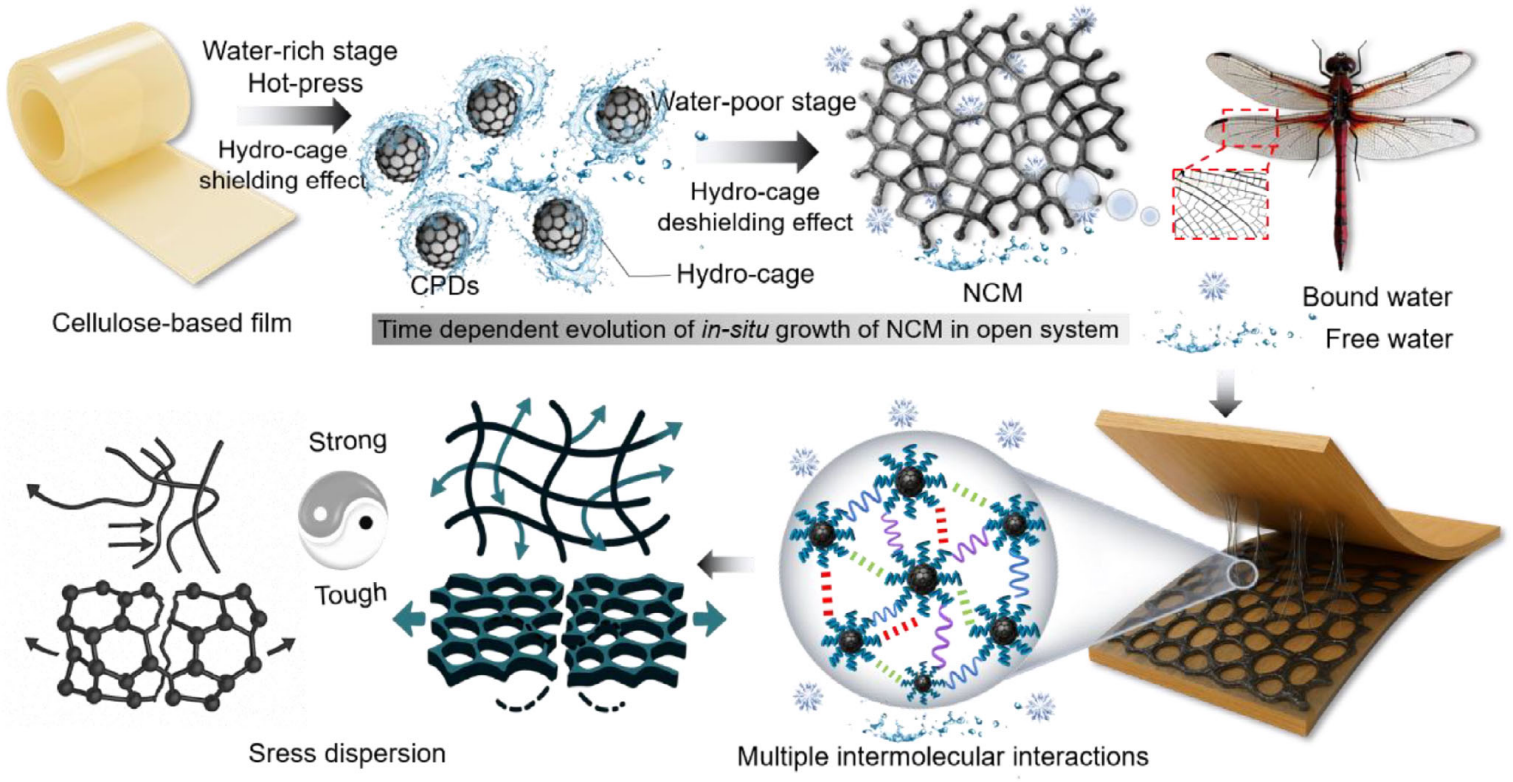
Conceptualizing an innovative “instantaneous carbonization‐polymerization” strategy for in situ growth to construct NCM with a dragonfly wing‐like structure.

One of the most promising directions for NCM preparation is the development of green synthesis strategies that significantly reduce the environmental footprint of NCM production [30, 31]. Herein, dialdehyde cellulose (DAC), phytic acid (PA), and glycerol were employed as feedstocks to fabricate a cellulose‐based film (CF). Glycerol served to retain moisture, eliminating the need for additional water during subsequent carbonization [32]. Due to the phosphoric acid functional groups, PA significantly catalyzes the formation of a subsequent NCM. During the initial stage of hot pressing, the CF gradually underwent carbonization and transformed into CPDs. Owing to the presence of abundant water, a hydro‐cage shielding effect formed between CPDs, inhibiting their further growth into NCM. In an open system, as hot pressing proceeded, water continuously evaporated or transitioned from a free to a bound state, gradually weakening the hydro‐cage shielding effect. Consequently, CPDs acted as nucleation cores and underwent further polymerization and growth via surface functional groups, ultimately forming a continuous NCM. The introduction of NCM significantly enhanced non‐covalent interfacial interactions, including van der Waals forces, electrostatic interactions, hydrogen bonding, and mechanical interlocking, thereby improving cohesion and bonding performance (Scheme 1). More importantly, the resulting NCM exhibited a hierarchical microstructure reminiscent of dragonfly wings, in which soft membrane regions dissipate stress energy while rigid vein‐like frameworks serve as reinforcing skeletons to transmit stress and resist mechanical deformation, endowing the NCM with excellent toughness and load‐bearing capacity [17, 18]. Importantly, the hydro‐cage de‐shielding strategy enabling instantaneous carbonization–polymerization avoids the high energy consumption and process complexity associated with conventional NCM synthesis, offering a green, low‐carbon, and scalable route toward economically viable applications.

### 2.1 Time Dependent Evolution of In Situ Growth of NCM

The microcrystalline cellulose (MCC) was selectively oxidized using sodium periodate to produce dialdehyde cellulose (DAC), then combined with phytic acid (PA) and glycerol to fabricate cellulose‐based film (CF) (Figures S1 and S2). Glycerol revealed excellent effect in enhancing plasticity and significantly improved the toughness of the film (Figure S3). In addition, glycerol also possessed a moisture‐retaining effect, which always enables the system to maintain a certain water content during the later carbonization process. (Figure S4) As‐prepared cellulose‐based film demonstrated exceptional mechanical properties (Figures S5–S8). Notably, the cellulose‐based film showed superior tensile strengths and Young's moduli in comparison with other widely used plastics (Figure S9) [33], revealing great potential for transportation and storage, which also provided a strong guarantee for subsequent carbonization process and large‐scale utilization.

**FIGURE 1:**
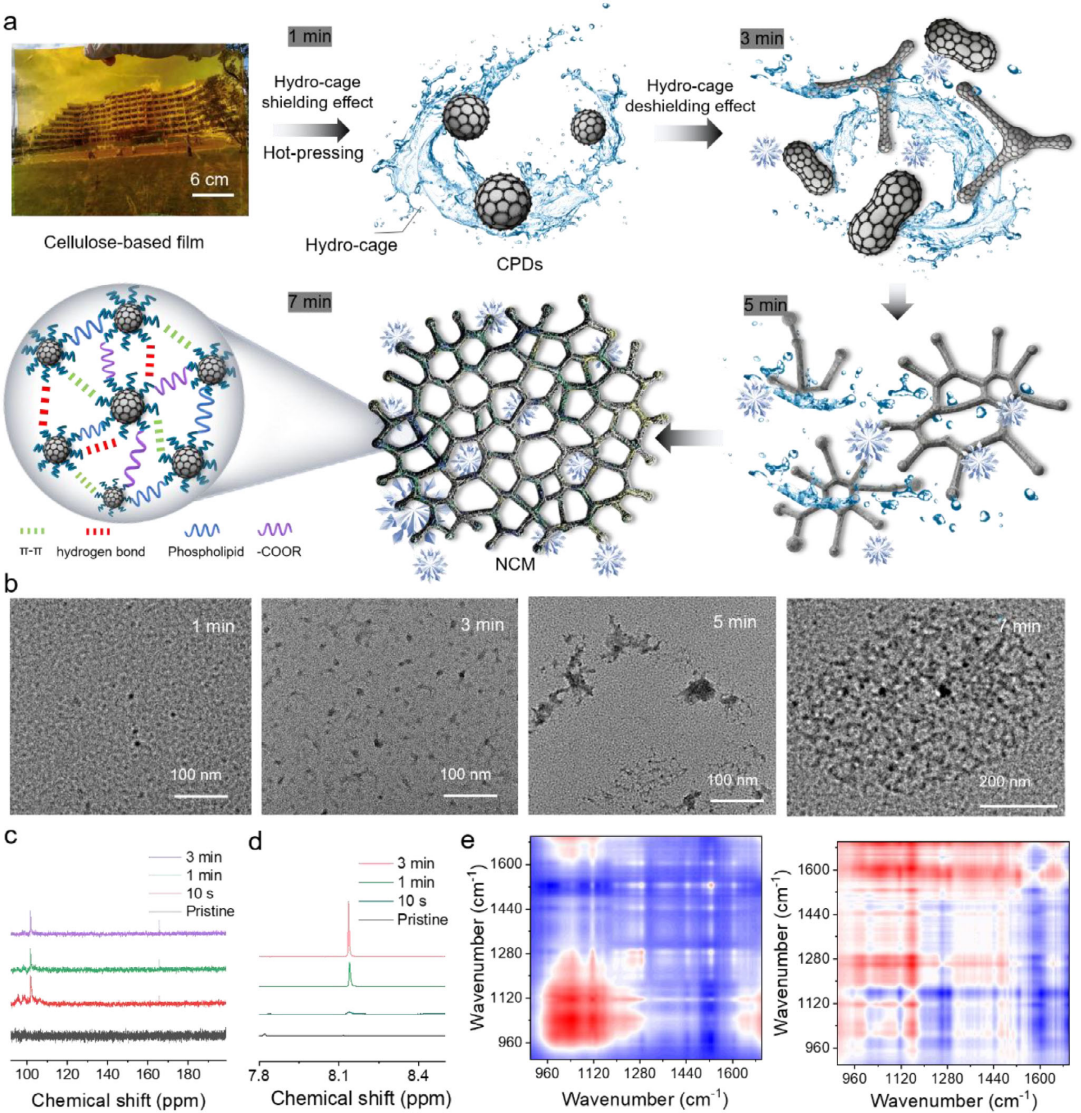
Time dependent evolution of in situ growth of NCM. (a)“Instantaneous carbonization‐polymerization” strategy for in situ carbonization to construct NCM with a dragonfly wing‐like structure by one‐step hot‐pressing process. (b) Time dependent in situ growth of NCM characterized by TEM after hot‐pressing for different times. (c) Time dependent in situ growth of NCM characterized by ^13^C NMR after hot‐pressing for different times. (d) Time dependent in situ growth of NCM characterized by ^1^HNMR after hot‐pressing for different times. (e) The 2D infrared correlation spectra of cellulose‐based film after hot‐pressing for different times. Left: 2D correlation synchronization graph; right: 2D correlation asynchronous graph. In 2D‐COS spectra, the warm color (red) represented positive intensities, while the cold color (blue) represented negative intensities.

During the initial stage of hot pressing, the CF gradually underwent carbonization and transformed into quasi‐spherical nanoparticles, namely CPDs. Here, the CF contained a large amount of water, with existed hydro‐cage shielding effect, which formed a huge barrier between CPDs, preventing CPDs from further polymerization and growing (Figure 1a,b). In open system, as the hot‐pressing progresses, water continuously evaporated or transformed from the free water state to the bound water state, the hydro‐cage shielding effect gradually weakened, where, the system formed a core of CPDs and further underwent polymerization and growth through its surface functional groups (e.g., ─CHO, ─OH, ─PO_3_H_2_, etc.), gradually forming NCM similar to a dragonfly's wing (Figure 1a,b). Notably, phytic acid plays an important catalytic role in the formation of the final NCM, directly determining the formation of CDPs and the carbon network. (Figure S10) [34]. Elemental mapping shows that different functional components were well dispersed in the NCM adhesive, which ensures the practical applicability of the adhesive. (Figure S11). High‐resolution images of NCMs revealed a clear lattice fringe of 0.21 nm, corresponding to the (100) crystal plane of graphitic carbon (Figure S12). Raman spectroscopy shown that after the hot‐pressing process, the degree of graphitization was significantly enhanced (Figure S13). This method eliminated the deficiency of traditional carbonization methods, significantly reducing energy consumption and process complexity, offering a new pathway for a green and low‐cost production of carbon‐based nanoparticles.

Small‐angle X‐ray scattering (SAXS) results revealed significant differences in the CF before and after hot‐pressing. As shown in Figure S14, the 2D scattering ring of cellulose‐based film before hot‐pressing was evenly distributed, indicating that the film primarily consisted of amorphous regions with low overall order, reflecting the limited size and loose arrangement of the structural domains. After hot‐pressing, the scattering ring showed higher brightness, indicating a reduction in the disordered structure, an increase in the correlation length, and the gradual formation of local ordered structures, confirming that the crystallinity and ordering of the structure were significantly improved. This change suggested that the hot‐pressing process induced carbonization and rearrangement of the organic structure within the film, transforming the system from a disordered aggregation state into a more compact and ordered structural network (Figures S15 and 16). Solid‐state 13C‐NMR (Figure S17) spectra showed signals at the 100–105 ppm region corresponding to hemiacetal structures, and a characteristic peak near 73 ppm corresponding to P─O─C bonds. The shift of peak value in the ^31^P spectrum (Figure S18) further verified the formation of phosphate bonds during hot pressing. The peaks near 100 and 165 ppm gradually strengthened, indicating that as the hot‐pressing process continued, the degree of graphitization gradually increased and carboxyl groups were also generated (Figure 1c). Furthermore, the signal of the phenolic hydroxyl group gradually increased around 8.1 ppm, which also indicated that as the hot‐pressing process continued, the graphitization gradually increased (Figure 1d). In XPS analysis, the C═O bond at 287.8 eV completely disappeared after hot‐pressing, indicating the occurrence of a hemiacetal reaction (Figure S19). Notably, the pronounced π–π satellite peak observed in the XPS C1s spectrum after hot pressing, together with the sharp G band and the relatively high IG/ID ratio in the Raman spectrum, jointly confirm that the carbon network synthesized via the hydro‐cage de‐shielding effect exhibits a high degree of graphitic crystallinity and an extended conjugated system. In general, the CPDs have grown into NCM through the synergistic effects of covalent bonds, e.g., phosphorylation, hemiacetal, and non‐covalent interactions, e.g., π–π conjugation and hydrogen bonds, etc. 2D infrared spectroscopy was conducted to reveal the dynamic evolution of cellulose‐based film after hot‐pressing for different times. As the hot‐pressing time increased, the π–π stacking interaction continued to strengthen. Although the hydrogen bonding gradually strengthened, its rate of change was slower than that of π–π stacking (Figure 1e).

### 2.2 The Influence of Hydro‐cage Shielding Effect for the Formation of NCM

**FIGURE 2:**
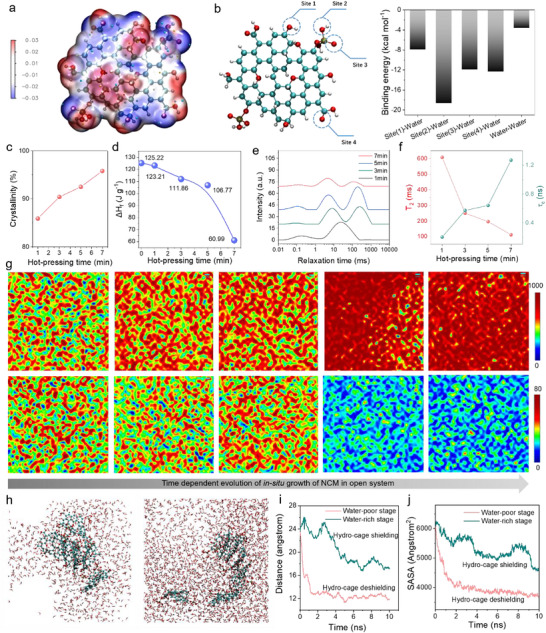
The influence of water content and state during different stages of hot pressing on the formation of NCM. (a) Van der Waals surface of the CPDs molecule colored by electrostatic potential. The scale bar was given in Hartree, with blue indicating negative electrostatic potential and red indicating positive electrostatic potential. Spherical markers on the surface denoted ESP extrema, with yellow indicating maxima and indigo indicating minima. Based on the ESP values and the positions of these extrema, several regions of high electrostatic potential were identified on the CPDs molecule, including the hydroxyl and aldehyde groups attached to the carbon ring, as well as the oxygen and hydroxyl atoms of the phosphate group. (b) Schematic illustration of the binding sites between the CPDs molecule and the water molecules. (c) The changes of crystallinity of cellulose‐based film after hot‐pressing for different times. (d) Enthalpy change of cellulose‐based film after hot‐pressing for different times. (e,f) The low‐field nuclear magnetic resonance (L‐NMR) analysis of cellulose‐based film after hot‐pressing for different times. (g) Time dependent in situ growth of nano carbon‐mesh characterized by Raman mapping after hot‐pressing for different times. Above: 1300–1750 cm^−1^, down: 1650–1850 cm^−1^. (h) Binding structures among CPDs molecules and between CPDs and water molecules under the low‐water condition (left) and high‐water condition (right). (i) Time evolution of the average intermolecular distance between CPDs. (j) Time evolution of the solvent‐accessible surface area (SASA) of CPDs.

The polar and nonpolar surface areas were calculated, where the Van der Waals surface of the CPDs molecule was colored by electrostatic potential (Figure 2a and Figure S16). The polar surface area was 502.09 Å^2^ (65.7% of the total surface area), while the nonpolar surface area was 262.13 Å^2^ (34.3% of the total), indicating that the molecule was overall polar. To quantitatively characterize the affinity of the high‐electrostatic‐potential sites on the CPDs molecule toward water, a water molecule was placed at each of the four lowest electrostatic potential sites identified in the ESP analysis. Geometry optimizations were then performed for each configuration, and the optimized structures are shown in Figures S20 and S21. It could be seen that the four sites of CPDs exhibit relatively strong binding with water, among which site 2 has the highest binding energy due to the formation of two hydrogen bonds with CPDs molecule (Figure 2b). Consequently, during the growth of CPDs, a gradual weakening of interaction with free water is accompanied by a corresponding enhancement of interaction with bound water, which further facilitated the hydro‐cage de‐shielding effect.

As the hot‐pressing time increased, the peak at ∼20.0° corresponding to the (101) crystal plane gradually increased, indicating that the formation of NCM significantly enhanced the crystallinity, which could increase internal dispersion, heterogeneity and bound water, leading to an ascension of crystallization, which strongly proved that the hydro‐cage de‐shielding effect was significantly enhanced. (Figure 2c). As the hot‐pressing process proceeds, the CPDs have gradually grown and transformed into NCM, where, NCM provided effective nucleation sites, reducing the required undercooling for crystallization and promoting an orderly arrangement of polymer chains. The surfaces of the NCM were rich in functional groups, which could interact with the polymer chains, further facilitating the formation and growth of crystallization nuclei, which increased the number and size of crystalline regions. Furthermore, the presence of NCM strengthened the interactions between the crystalline and amorphous regions, leading to a more stabilized network structure and a reduced enthalpy change with increasing hot‐pressing time. This structural stabilization is conducive to enhanced cohesion and bonding performance (Figure 2d). During hot‐pressing, the ratio of free water to bound water was constantly adjusted and reached an optimal value, subsequently regulating its cohesion. To verify the increase in bound water of cellulose‐based film after hot‐pressing for different times, low‐field nuclear magnetic resonance (L‐NMR) tests were employed to confirm the highly bound state of water in these hydrogels (Figure 2e,f). The proton spin‐spin relaxation time (T_2_) of water in cellulose‐based film after hot‐pressing for different times significantly decreased from 608.22 to 110.975 ms, besides, the τ_c_ increased from 0.2 to 1.1 ns, suggesting a decrease in the mobility of water molecules. This could be attributed to the enhanced strong hydrogen bonding restricting the mobility, resulting in an increase in bound water and a decrease in the mobility of remaining free water. The formation of NCM relied on the in situ growth of CPDs synchronously supporting the evolution of structure from loose disordered to dense ordered during hot pressing, which also explained the improvements in hydro‐cage de‐shielding effect and subsequent water resistance and bonding performance.

Raman mapping analysis revealed that with ongoing carbonization, the intensity of the D peak (∼1350 cm^−^^1^) and G peak (∼1580 cm^−^^1^) significantly increased, indicating improved orderliness of the graphitized carbon structure and enhanced π–π stacking (Figure 2g). Additionally, the intensity of the C─O vibration peak (1350–1450 cm^−^^1^) increased gradually as carbonization progress continued, suggesting that hydrogen bonding enhanced in the early carbonization stages and stabilized in the later stages. As the hot‐pressing time prolonged, the intensity of the C═O characteristic peak (∼1720 cm^−^^1^) decreased, while the C─O vibration signal in the 1300–1750 cm^−^^1^ range correspondingly increased, confirming that the aldehyde group gradually converted into C─O moieties, a process closely related to dehydration and condensation during carbonization. The above spectral evolution indicated that as the carbonization time increased, the cellulose‐based film underwent a transition from a rich aldehyde, weakly ordered state to a highly aromatic, highly crosslinked structure, with π‐π stacking and hydrogen bonding synergistically regulating the formation and stabilization of NCM.

Thermal transport measurements provide quantitative support for the structural and mechanistic evolution associated with the transition from the hydro‐cage shielding to the de‐shielding state (Tables S2 and S3). In the pre‐curing stage, the cellulose‐based precursor film (CF) exhibited pronounced dependence of thermal conductivity, thermal diffusivity and specific heat capacity on the applied heat flux power and rate. Under high‐power pulse conditions, the apparent thermal conductivity increased by more than twofold compared with that measured under low‐power conditions, accompanied by a significant decrease in specific heat capacity. Such highly sensitive and unstable thermal transport behavior is consistent with a metastable structure rich in free water, in which water molecules form dynamic hydro‐cage environments around the initial CPDs, interrupting continuous heat‐transfer pathways. Transient thermal perturbations may induce water redistribution and local structural rearrangements, resulting in substantial but non‐intrinsic fluctuations in macroscopic thermal parameters.

By contrast, after standard hot‐pressing treatment, the thermal transport behavior of the material underwent a marked transition. The resulting nano carbon‐mesh (NCM) displayed thermal conductivity, thermal diffusivity, and specific heat capacity that were no longer influenced by measurement conditions, instead exhibiting stable intrinsic responses as a function of temperature. Notably, the thermal conductivity of NCM increased monotonically from 0.080 W m^−^^1^ K^−^^1^ at 25°C to 0.113 W m^−^^1^ K^−^^1^ at 200°C, indicating that heat transport became dominated by a continuous and stable network structure. Concurrently, the pronounced increase in specific heat capacity suggested an enhanced ability of the system to store and regulate thermal energy, while the stabilization of thermal diffusivity at medium and high temperatures further supported the formation of efficient heat‐transfer pathways. The overall transition in thermal transport properties was consistent with the progressive evaporation of water and the conversion of free water to bound water during hot pressing, supporting the effective attenuation of the hydro‐cage shielding effect and the in situ polymerization of CPDs via covalent and non‐covalent interactions to form a continuous NCM skeleton.

It could be observed that CPDs molecules aggregated more tightly in the presence of a limited amount of water, whereas their aggregation became more diffuse when water was abundant, which dynamically confirmed that intermolecular interactions among CPDs and between CPDs and water molecules in the water‐poor stage were more intense (Figure 2h). To further quantify the aggregation behavior of CPDs during the molecular dynamic simulations, the intermolecular distances between CPDs were calculated for both the low‐water and high‐water conditions. It was clearly observed that the average intermolecular distance under the low‐water condition was approximately half of that under the high‐water condition, indicating that CPDs molecules aggregate much more tightly when water was limited (Figure 2i). To further quantify the association between CPDs molecules, the solvent‐accessible surface area (SASA) of the CPDs was calculated. SASA reflected the degree of solvent aggregation, with smaller SASA values generally indicating tighter solvent binding. The total SASA of six CPDs molecules was computed for the two scenarios, and the results are shown in Figure 2j. Consistent with the analysis of inter‐CPDs distances, in the presence of a small amount of water, the SASA of CPDs decreased significantly, indicating a tighter association among the CPDs molecules. As the hot‐pressing process continues, the hydro‐cage de‐shielding effect intensified, the degree of graphitization also increased, resulting the hydrophobicity gradually enhanced, thereby facilitating the conversion of CPDs to NCM.

### 2.3 Bonding Performance of NCM

**FIGURE 3:**
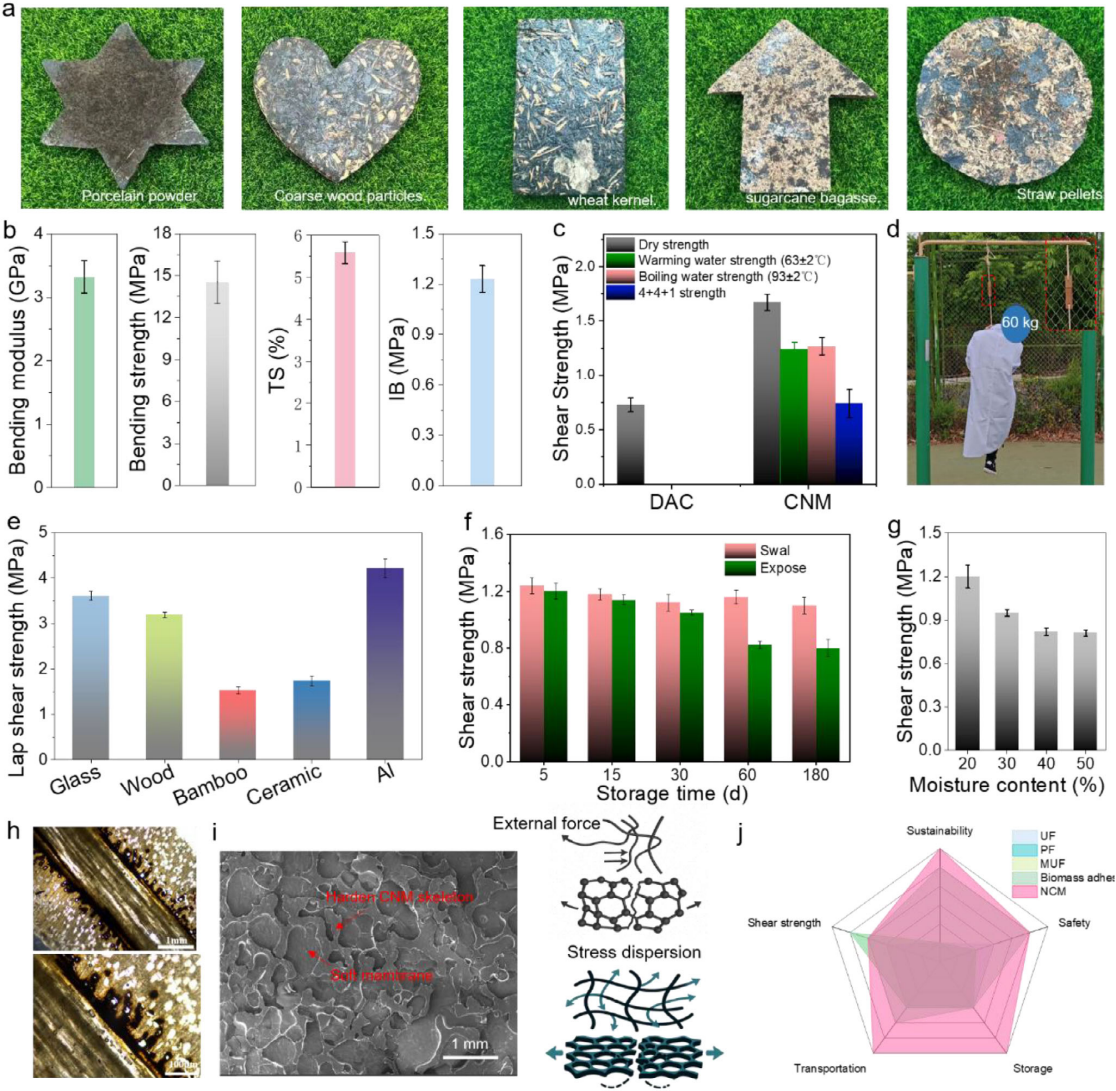
Bonding performance of NCM. (a) NCM for different substrates’ shape regulation. (b) The static bending modulus, bending strength, TS, and internal bond strength. (c) Dry strength, hot water strength, and boiling water strength of three‐layer plywood under the different content of NCM. (d) Demonstration of plywood supporting a 60 kg load. (e) Lap shear strength of NCM on various substrates. (f) Bonding strength of plywood after depositing for different times (Both sealed and exposed film samples were stored in a constant temperature and humidity chamber at 30°C and 40% humidity). (g) Bonding performance at different moisture contents of plywood. (h) Optical microscope view of plywood cross‐section. (i) Microscope view of plywood cross‐section. (j) The superiority of the novel NCM.

This work employed hydro‐cage de‐shielding strategy to implement instantaneous carbonization‐polymerization to fabricate NCM showing excellent bonding performance. Interestingly, this strategy demonstrated broad substrate compatibility, achieving strong adhesion with coarse wood particles, bagasse, wheat straw, and rice straw (Figure 3a). Importantly, this study also explored the application of recycled cellulose‐based films in chipboard. Recycled film powder was mixed with wood particles, then hot‐pressed into composite panels (Figure 3b), which fully met the requirements for P3‐grade panels according to GB/T 4897‐2015: internal bonding (IB) strength of 1.23 MPa, modulus of rupture (MOR) of 14.5 MPa, and a low 24 h thickness swelling (TS) of 5.58%.

The research revealed that pure cellulose film was unable to successfully adhere to the substrates, but the NCM possessed excellent bonding performance. Using poplar veneers as the substrate, three‐layer plywood was successfully fabricated by hot‐pressing (Figure 3c). The bonding performance of plywood prepared with NCM was systematically evaluated. The wet shear strength of the NCM‐plywood exhibited a significantly enhanced strength of 1.24 ± 0.05 MPa after immersion in hot water at 63°C for 3 h, far exceeding the GB/T 9846‐2015 requirement for Class II plywood (≥ 0.7 MPa). Even under the more rigorous boiling‐water cycle treatment, the NCM‐plywood maintained a strength of 0.73 ± 0.04 MPa, meeting the Class I board standard, whereas the control groups (DAC) completely failed. More importantly, the bonding performance gradually increased with increasing hot‐pressing time, which demonstrated that the formation of the carbon network makes a significant contribution to the bonding strength. (Figure S22). Failure mode analysis further confirmed the interfacial strengthening mechanism: fracture surfaces of NCM specimens were dominated by fiber tearing (> 80% matrix failure) with no visible interfacial debonding, indicating that the interfacial bonding strength surpassed that of the wood substrate itself (Figure S23).

A plywood specimen after hot‐pressing treatment (bonding area: 20 × 25 mm) exhibited sufficient tensile strength to support the weight of adults (approximately 60 kg) (Figure 3d). The poplar‐based, bamboo‐based, aluminum‐based, ceramics‐based, and glass‐based substrates underwent hot‐pressing, and the results showed bonding strengths in the order of aluminum (4.21 MPa) > glass (3.61 MPa) > poplar (3.19 MPa) > ceramics (1.74 MPa) > bamboo (1.59 MPa), which confirmed that recycled cellulose‐based film was carbonized into NCM after hot‐pressing, and could achieve robust bonding performance across a wide range of substrates (Figure 3e and Figure S24). The long‐term evolution of wet shear strength under sealed and exposed storage conditions was systematically investigated. Under sealed storage, the wet shear strength (1.24 MPa) remained nearly constant after 180 days. Under exposed conditions, the strength remained at 0.83 MPa, still meeting the national standard (≥ 0.7 MPa) (Figure 3f). In contrast, conventional urea‐formaldehyde (UF) adhesives readily underwent premature curing, leading to short shelf life and decrease in the bonding performance. When the moisture content was 20%, 30%, 40%, and 50%, the wet shear strength of the NCM‐plywood was 1.24, 0.95, 0.82, and 0.91 MPa, respectively, exceeding the national standard (≥ 0.7 MPa). Even under the extreme condition of 50% moisture content, effective bonding was maintained, whereas plywood bonded with UF resin completely failed, highlighting its unique application advantage in humid environments (Figure 3g).

Inspired by the multi‐scale structure of the “rigid veins–soft wing membrane” of dragonfly wings, a biomimetic enhancement mechanism was proposed in this study, where the in situ generated NCM during hot‐pressing acted as the “rigid vein framework,” effectively transmitting and dispersing stress through strong π–π conjugation, phosphate, and hemiacetal moieties, forming a continuous and stable three‐dimensional network. The embedded cellulose functionalized as the “soft tissue,” primarily buffering external impacts through hydrogen bond reconstruction, molecular chain sliding, and energy dissipation (Figure 3h,i, and Figures S25 and S26). Generally, the cellulose‐based film before carbonization enabled convenient storage and transportation of solid‐state adhesive films (with a shelf life exceeding 180 days). Generally, employing hydro‐cage de‐shielding strategy to implement instantaneous carbonization‐polymerization to construct the composite, endowed the composite with excellent storage and transportation capabilities as well as superior bonding performance, which was conducive to the sustainable development of composites (Figure 3j) [35, 36, 37, 38].

Concurrently, cone calorimeter analysis revealed that NCM exhibited a total heat release (THR) of 31.98 MJ m^−^^2^, 36.8% lower than UF resin (50.63 MJ m^−^^2^). The total smoke release (TSR) was 67.33 m^2^ m^−^^2^, lower than UF resin (70.79 m^2^ m^−^^2^). The mass loss of NCM‐1.0 was 2025.30 g m^−^^2^, lower than that of UF resin (2574.08 g m^−^^2^). The comprehensive results indicated that plywood laminated with NCM effectively delayed ignition, suppressed combustion and reduced smoke release compared to UF resin (Figures S27–S29 and Table S4). To further evaluate the emission safety of NCM‐based binder systems, the volatile organic compounds (VOCs) released during their thermal decomposition were analyzed and compared with those from conventional urea‐formaldehyde (UF) resins. As shown in Figures S30 and S31, the complexity of NCM pyrolysis products was significantly reduced, with only eight suspected identifiable VOCs species detected across the studied temperature range. In contrast, UF resin released approximately forty distinct VOCs under identical pyrolysis conditions, indicating substantially higher complexity in its volatile degradation products. These results demonstrated that NCM‐based adhesives not only exhibited excellent mechanical strength and moisture resistance but also showed significantly reduced complexity in volatile organic compound release under pyrolysis and combustion‐related conditions. This reflected superior thermal stability and emission safety characteristics, providing robust assurance for the safety of wood‐based composites during use and indoor air quality.

## 3 Conclusion

NCM has shown great potential in many fields, but traditional methods for synthesizing NCM require high temperature and pressure, complex processes, inferior reproducibility, leading to enormous energy consumption, making it difficult to achieve green, low‐carbon, and large‐scale applications. Developing a new method for synthesizing NCM that is green and conducive to large‐scale production is the current mainstream. In this work, cellulose‐based film (CF) was gradually carbonized into quasi‐spherical CPDs at the initial stage, here, system contained enormous water, existed hydro‐cage shielding effect, which formed a huge barrier between CPDs, preventing CPDs from further polymerization and growing. As the hot‐pressing progresses, water continuously evaporated or transformed from the free water state to the bound water state, the hydro‐cage shielding effect gradually weakened, the system formed a core of CPDs and further underwent polymerization and growth through its surface functional groups (e.g., ─CHO, ─OH, ─PO_3_H_2_, etc.), gradually forming NCM similar to a dragonfly's wing consisting of soft polymer membranes and rigid veins, where the rigid veins serve as a reinforcing skeleton to transmit stress and resist mechanical deformation, while the surrounding soft membranes also played a key role in stress energy dissipation. This highly organized hierarchical structure and unique fracture mechanism endowed NCM with exceptional bonding performance and load‐bearing capacity.

## 4 Experimental Section

### 4.1 Materials

Microcrystalline cellulose (MCC, 97%) was purchased from Shanghai Yuanye Biotechnology Co., Ltd. Sodium periodate (NaIO_4_, AR 99.5%) and phytic acid (PA, 70%) were purchased from Shanghai McLean Biochemical Technology Co., Ltd. Glycerol (AR) was purchased from Sinopharm Chemical Reagents Co., Ltd. Poplar veneer (moisture content approximately 10–12%), dimensions 1270 × 840 × 2 mm, was purchased from Heze Junan Wood Industry Co., Ltd.

### 4.2 Preparation of Dialdehyde Cellulose (DAC)

The microcrystalline cellulose (24.0 g) was dispersed in deionized water (1000.0 g). The flask was carefully wrapped using aluminum foil to prevent light from inducing the decomposition of sodium periodate. The sodium periodate (38.0 g) was dissolved in deionized water, then added into above cellulose suspension at 48°C and stirred at 800 rpm. After 19 h, the product was washed with deionized water by centrifugation until the pH value reached pH of 7, the centrifuged solid was removed and freeze‐dried to obtain the DAC.

### 4.3 Preparation of Cellulose‐based Film

Table S1 shows the different formulations of the cellulose‐based film. The DAC suspension was stirred for 1 h. After the solution clarified, phytic acid (PA) was added to the DAC suspension, and the reaction continued stirred for 30 min. Then, glycerol was added, and the reaction was completed after another 30 min. The solution was poured into a mold and air‐dried to form the cellulose‐based film. The dried cellulose‐based film was placed in a constant temperature and humidity chamber for three days to equilibrate its moisture content (30%).

### 4.4 Evaluation of the Oxidation Degree (OD) of DAC

The oxidation degree (OD) of DAC was determined using titration and elemental analysis methods. The 10 mL of DAC solution with a pH of 3.5 was mixed with 10 mL of 5% hydroxylamine hydrochloride solution and titrated with 0.05 m NaOH to a pH of 3.5 at room temperature using the titration analysis method. Finally, the OD of DAC was calculated using the following formula:

(1) $$\mathrm{OD}=\frac{162\times C_{\mathrm{NAOH}}V_{\mathrm{NAOH}}}{m+2\times C_{\mathrm{NAOH}}V_{\mathrm{NAOH}}}$$

where, OD represented the degree of oxidation of DAC, 162 represented the molecular weight of the glucose groups in cellulose, C_NaOH_ and V_NaOH_ represented the concentration and volume of the sodium hydroxide standard solution, and m represented the dry weight of DAC.

### 4.5 Other Measurements

#### 4.5.1 Mechanical Performance Investigation

On the SUST 5569 universal testing machine, samples were cut into rectangles measuring 150 mm in length and 15 mm in width. Tensile tests were conducted at a speed of 50 mm min^−1^ under conditions of 25°C room temperature and ∼50% relative humidity. Notably, five repeated tests were conducted on each sample.

#### 4.5.2 Raman Spectroscopy Analysis

Raman spectra of the samples were acquired using a Thermo Fisher DXR 2xi confocal Raman spectrometer with a 532 nm laser at 5 mW laser intensity.

#### 4.5.3 Small‐angle X‐ray Scattering (SAXS) Measurements

SAXS measurements were conducted using an Anton Paar SAXSpoint2.0 with a Cu target (Kα radiation, λ = 0.154184 nm) and an X‐ray wavelength (λ) of 1.53 nm. The sample‐to‐detector distance was 300 mm, and the signal was collected using a 2D hybrid photon counting detector (EIGER R 1M). The scattered data were analyzed using Fit 2D software, and the SAXS 2D patterns were integrated over a fan‐shaped region to obtain the one‐dimensional scattering profile. The scattering vector (q) was calculated using the equation:

(2) $$q=\frac{4\pi \sin\theta }{\lambda }$$

where λ was the X‐ray wavelength and θ was the scattering angle. The one‐dimensional curve was integrated, and the average distance between adjacent crystalline domains was then calculated using Bragg's law:

(3) $$L=\frac{2\pi }{q_{\max}}$$

where qmax was the value of q at the maximum scattering intensity.

#### 4.5.4 Low‐field Nuclear Magnetic Resonance (NMR) Analysis

The proton spin‐spin relaxation time (T2) of the hydrogel was measured using a MesoMR23‐060H‐I nuclear magnetic resonance imaging analyzer, with a proton resonance frequency of 21 MHz (0.5 T), a resonance frequency of 23 MHz, a coil diameter of 25 mm and a magnet temperature of 32°C. The correlation time (τc) of water molecular motion was calculated using the Bloembergen‐Purcell‐Pound equation, thus enabling the quantitative analysis of water dynamics:

(4) $$\frac{1}{T_{2}}=\frac{1}{T_{2}}\left(3\tau_{c}+\frac{5\tau_{c}}{1+\omega_{0}^{2}\tau_{c}^{2}}+\frac{2\tau_{c}}{1+4\omega_{0}^{2}\tau_{c}^{2}}\right)$$

Here, C was the water constant (5.33 × 109 S^−2^), and ω0 is the Larmor frequency.

#### 4.5.5 Transmission Electron Microscope (TEM) Analysis

TEM images were acquired using a transmission electron microscope and the FEI Talos F200S (USA) at an accelerating voltage of 200 kV.

#### 4.5.6 Plywood Preparation and Testing of Mechanical Properties

The cellulose‐based film was uniformly sandwiched between the two sides of the central veneer at an adhesive application rate of 270 g m^−2^. The panels were hot‐pressed under temperature of 200°C and pressure of 1 MPa for 7 min, resulting in three‐layer plywood samples with dimensions of 120 mm × 190 mm × 2 mm. According to GB/T 17657‐2022, six samples were cut and taken from each panel, with a bonding area of 25 mm × 25 mm. The shear strength of the specimens was calculated according to the method specified in GB/T 17657‐2022. The dry shear strength and wet shear strength values of the samples were determined by immersing them in hot water for 3 h (63 ± 2°C), boiling water for 3 h (93 ± 2°C), and boiling water cycle immersion (GB/T 17657‐2022), respectively, to evaluate the adhesive performance of the adhesive. The strength values mentioned in this paper represented the average of six repeated measurements for each test.

#### 4.5.7 Fourier Transform Infrared Spectroscopy (FT‐IR)

The interactions between the various components of the film were studied using a Nicolet IS 50 attenuated total reflection infrared spectrometer. Each sample was examined in the range of 4000–400 cm^−1^ with a resolution of 4 cm^−1^ and scanned a total of 32 times.

#### 4.5.8 NMR Analysis

The interactions between the various components of films before and after hot pressing were studied using solid‐state 13NCMR and 31PNMR spectroscopy with an AVANCE II 400 MHz spectrometer (Brüker, Billerica, MA, USA), using a 4 mm probe at 12 kHz sample rotation.

#### 4.5.9 X‐ray Photoelectron Spectroscopy (XPS) Analysis

The cellulose‐based film was analyzed using an XPS instrument (Thermo, USA) to determine the changes of binding energy before and after hot pressing. The full spectrum flux energy was 100 eV, and the narrow spectrum flux energy was 50 eV. The C1s peak of surface unstable carbon was calibrated at a binding energy of 284.8 eV, and the spectrum was processed using Advantage analysis software.

#### 4.5.10 Differential Scanning Calorimetry (DSC) Analysis

DSC analysis was performed using a DSC Q20 calorimeter (TA Instruments, USA) at a heating rate of 10°C min^−1^ from 30°C to 250°C to analyze the thermal curing behavior of the films.

#### 4.5.11 Scanning Electron Microscopy (SEM) Analysis

The specimens were cut into small pieces (3 mm × 4 mm × 3 mm) using a single‐edged razor blade, and the surface of each small piece was polished using a sliding microtome. After coating with approximately 10–20 nm of gold, the surface morphology of the adhesive line area of the lap test specimens was examined using SEM (Thermo Scientific, USA).

#### 4.5.12 The Images of the Gluing Line of the Bonding Specimens

The samples were cut into sections using an ultra‐thin sectioning machine (LEICA SM 2000R). The soluble impurities were removed by rinsing with deionized water, then dried and fixed on a glass slide. Obtain images of the bond lines of the specimen using an electron microscope (Nikon eclipse 80i DM 2000).

#### 4.5.13 Shear Strength Testing of Specimens with Different Moisture Contents

The initial moisture content of wood veneers (190 mm in length, 120 mm in width, and 2 mm in thickness) stored at room temperature and atmospheric pressure was determined using a moisture meter (MB 90, OHAUS), yielding a result of 6 ± 0.36%. The wood veneer was then completely immersed in distilled water for 12 h and subsequently placed in a constant temperature and humidity chamber (HWS‐250, 23°C, 60% RH) for drying. The samples were removed after 1 h, 1 h 30 min, 2 h 15 min, 3 h, 3 h 30 min, and 5 h, and their moisture contents were measured at 50.21 ± 2.65%, 40.61 ± 1.82%, 29.68 ± 1.40%, and 20 ± 2.02%, respectively. The wet shear strength of the three‐layer plywood specimens was tested using the same method as described above (with a one‐minute pre‐pressing step during the pressing of the plywood).

#### 4.5.14 Adhesion Test

The adhesion strength of the sample to various substrates was evaluated through lap strength testing. Yang wood‐based panels were assembled with a width of 25 mm, a length of 90 mm, and a thickness of 3 mm. The film was uniformly applied to the core layer with an overlap area of 25 mm × 25 mm.

Bamboo‐based bamboo strips were assembled with a width of 20 mm, a length of 90 mm, and a thickness of 3 mm. The film was uniformly applied to the core layer with an overlap area of 20 mm × 25 mm. Other materials such as aluminum sheets, glass, and ceramic sheets, were assembled with dimensions of 25 mm in width, 90 mm in length, and 3 mm in thickness. The film was uniformly applied to the core layer with an overlapping area of 20 mm × 25 mm.

The poplar‐based wood panels and bamboo strips were hot‐pressed at 200°C, 1 MPa, and 7 min. Other materials were baked at 200°C for 3 h to assess the adhesive strength of the film on the substrate. The shear strength was measured using a universal testing machine at a speed of 15 mm min^−1^ through a lap shear test and calculated using the following formula:

(5) $$\mathrm{Adhesive}\;\mathrm{strength}\;=\frac{\mathrm{maximum}\;\mathrm{force}\;\left(N\right)}{\mathrm{Bonding}\;\mathrm{area}\;\left(m^{2}\right)}$$

### 4.6 Computational Calculation

#### 4.6.1 Geometry Optimization of Quantum Chemical Calculations

Quantum chemical calculations were performed using the ORCA [39]software package. Geometry optimization of an isolated CPDs molecule was carried out using density functional theory (DFT) with the B3LYP functional, the def2‐SVP basis set, D3 dispersion correction, and the auxiliary basis set (RI) enabled. The optimized molecular geometries were optimized and were visualized using the VMD [40] software.

#### 4.6.2 Electrostatic Potential of Quantum Chemical Calculations

To evaluate the ability of CPDs to interact with water molecules, van der Waals surfaces colored by electrostatic potential were generated and analyzed using the Multiwfn [41, 42] program.

#### 4.6.3 Binding Energy of Quantum Chemical Calculations

To quantitatively characterize the affinity of the high‐electrostatic‐potential sites on the CPDs molecule toward water, a water molecule was placed at each of the four lowest electrostatic potential sites identified in the ESP analysis. Geometry optimizations were then performed for each configuration. Subsequently, the binding energies between the water molecule and the CPDs molecule were calculated. The binding energy is defined as follows:

(6) $$E_{binding\;energy}=E_{complex}\;-E_{CPDs}-E_{H_{2}O}$$

In this equation, *E_complex_* denotes the single‐point energy of the CPDs–water complex, *E_CPDs_* is the single‐point energy of the CPDs molecule in the complex, and $E_{H_{2}O}$ is the single‐point energy of the water molecule in the complex.

#### 4.6.4 Molecular Dynamic Simulation

Molecular dynamics simulations were performed using the LAMMPS [39, 43] software package. Two conditions were considered: a low‐water environment and a high‐water environment. The simulation box size was 50 × 50 × 50 Å. In the low‐water case, 1000 water molecules were added to the box, whereas 2500 water molecules were added in the high‐water case. In both scenarios, six CPDs molecules were included. The simulations were carried out using the CVFF force field in the NVT ensemble at a temperature of 300 K.

The average distance between CPDs was defined as the mean distance from one CPDs molecule to the other five CPDs molecules.

## Conflicts of Interest

The authors declare no conflict of interest.

## Supporting information




**Supporting File**: advs75630‐sup‐0001‐SuppMat.docx.

## Data Availability

The data that support the findings of this study are available in the supplementary material of this article.
