# Supplementary material for: Nano Carbon‐mesh with Excellent Bonding Performance via Hydro‐cage De‐shielding Strategy
Source: Adv Sci (Weinh). 2026 May 13:e75630. Online ahead of print. doi: 10.1002/advs.75630 (PMC13336100; doi:10.1002/advs.75630)
Supplement: Supplementary file 1 — Supporting file 1: advs75630‐sup‐0001‐SuppMat.docx [file ADVS-9999-e75630-s001.docx]

**Supporting Information**

**Nano Carbon-mesh with Excellent Bonding Performance via Hydro-cage De-shielding Strategy**

Weijia Yang,^[a]^ Jianyong Wan,^[a]^ Hongda Guo,^[a]^ Haizhu Wu,^[a]^ Yongli Yang,^[a]^ Wenhe Bi,^[a]^ Zhengyong Yang,^[a]^ Chenghua Wang,^[a]^ Bertrand Charrier,^[b]^ Hisham Essawy,^[c]^ Antonio Pizzi,^[d]^ Xinyi Chen,^[a]^ Zhijun Chen,*^[e]^ Guanben Du*,**^[a]^ Xiaojian Zhou*^[a]^

1. Yunnan Provincial Key Laboratory of Wood and Bamboo Biomass Materials

Southwest Forestry University

Kunming 650224 (China)

E-mail: [guanben@swfu.edu.cn](mailto:guanben@swfu.edu.cn); [xiaojianzhou@swfu.edu.cn](mailto:xiaojianzhou@swfu.edu.cn)

1. CNRS/Univ Pau & Pays Adour, Institut des Sciences Analytiques et de

Physico -Chimie pour lEnvironnement et les Matériaux-Xylomat, UMR5254

40004 Mont-de-Marsan (France)

1. National Research Centre, Department of Polymers and Pigments Cairo, 12622, Egypt
2. LERMAB, University of Lorraine, 27 rue Philippe Seguin 88000, Epinal, France

Key Laboratory of Bio-based Material Science & Technology, Northeast Forestry University, Ministry of Education,

Harbin 150040 (China)

E-mail: [chenzhijun@nefu.edu.cn](mailto:chenzhijun@nefu.edu.cn)

**Table of Contents**

S1. The basic properties of the cellulose-based film 2

[S2. Characterization of nano carbon -mesh materials 3](#_TOC_250004)

[S3. Tables 12](#_TOC_250003)

# S1. The basic properties of the cellulose-based film.


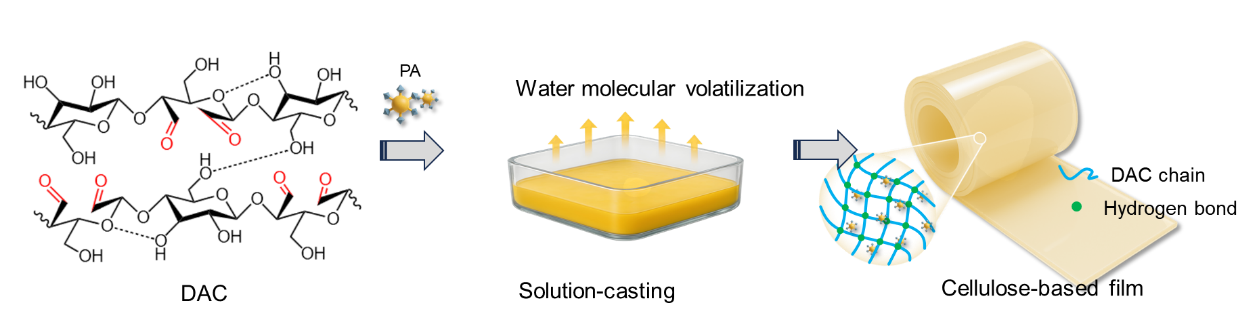


**Figure S1.** Preparation of cellulose-based film (CF)


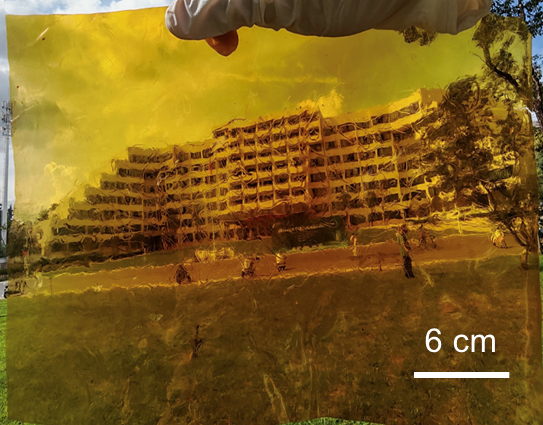


**Figure S2.** The display of cellulose-based film.


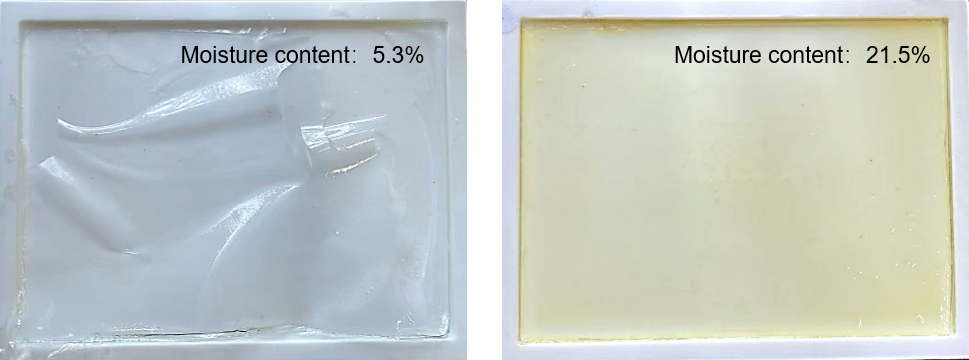


**Figure S3.** Film formation comparison (Left: without glycerin; Right: with glycerin)

.

# S2. Characterization of nano carbon -mesh materials


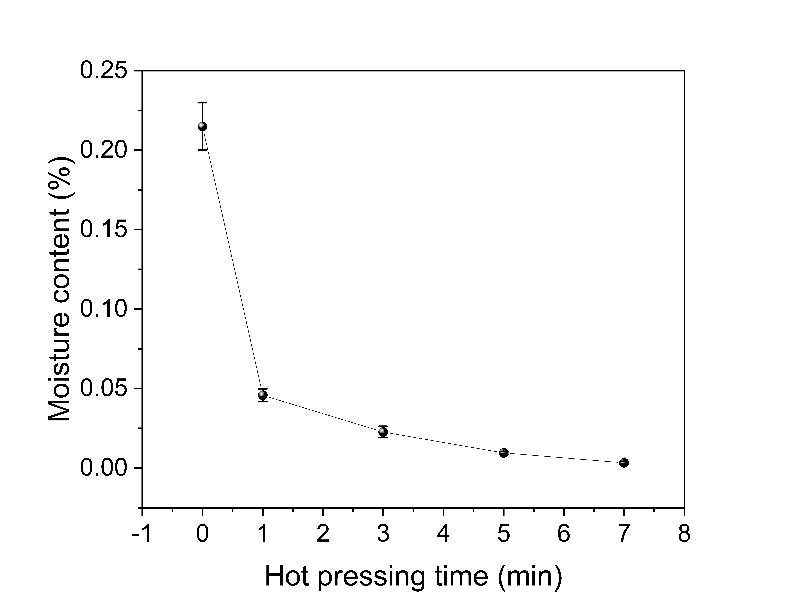


**Figure S4.** Changes in moisture content during the hot-pressing process.

.


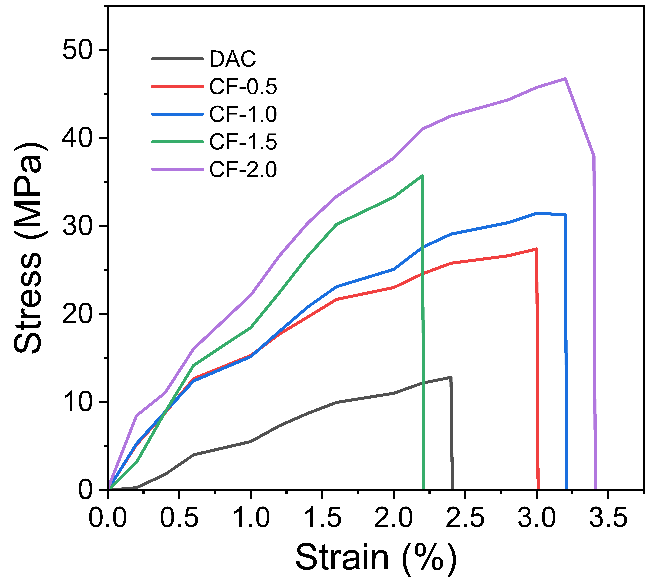


**Figure S5.** Stress-strain curves of CF.


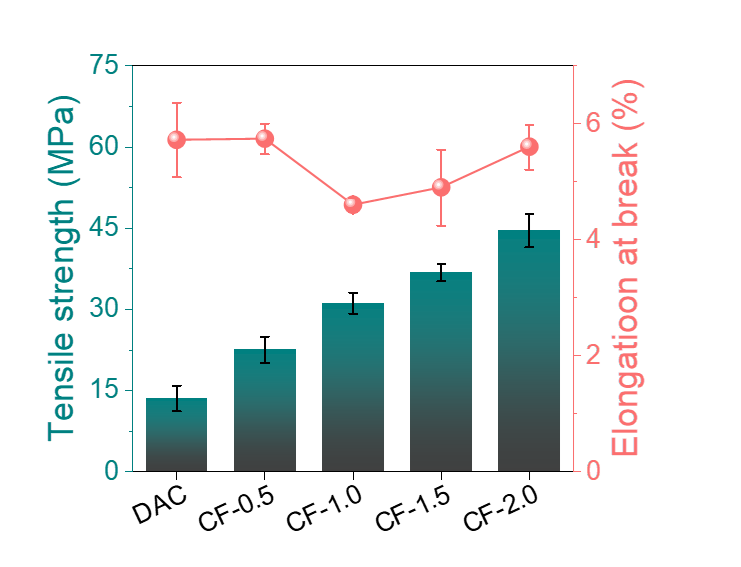


**Figure S6.** Tensile strength and elongation at break of CF.


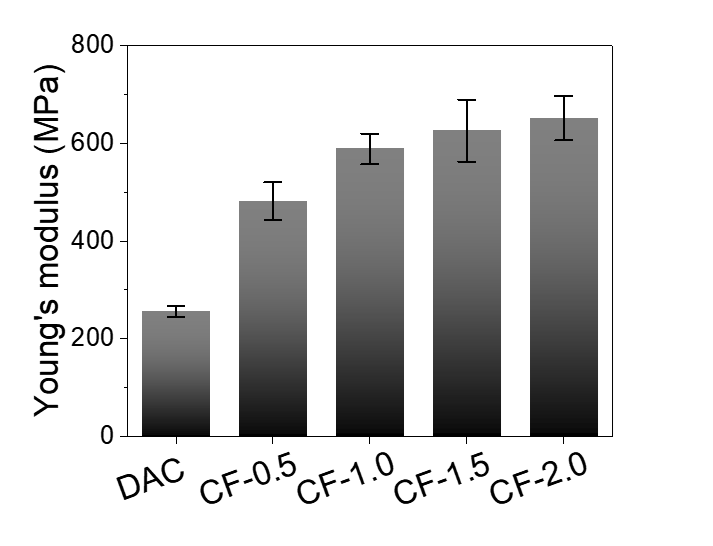


**Figure S7.** Young’s moduli of cellulose-based film (CF).


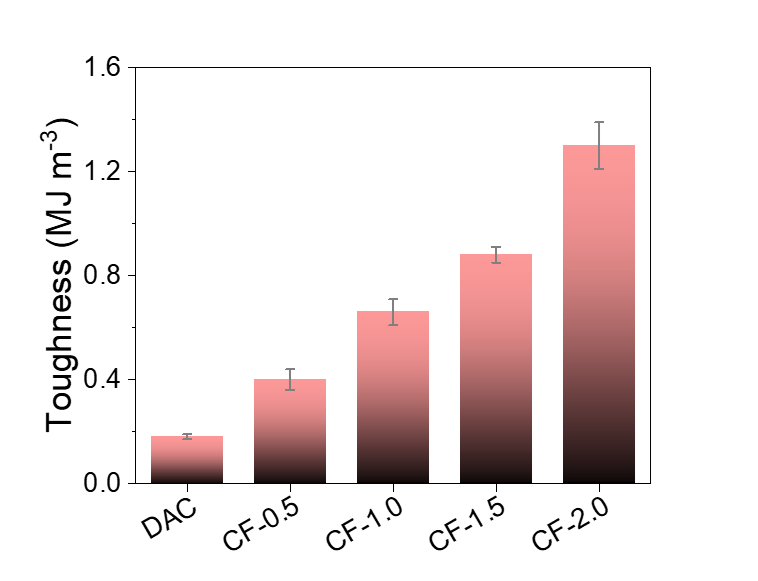


**Figure S8.** Toughness of cellulose-based film (CF).


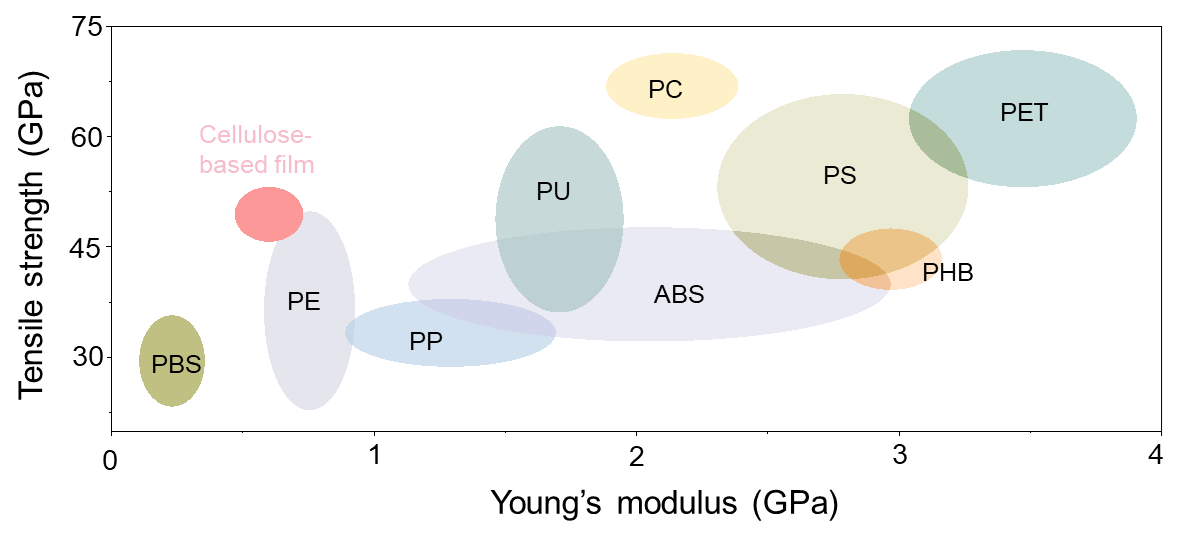


**Figure S9.** Tensile strengths and Young’s moduli of CF compared with currently widely used plastics.


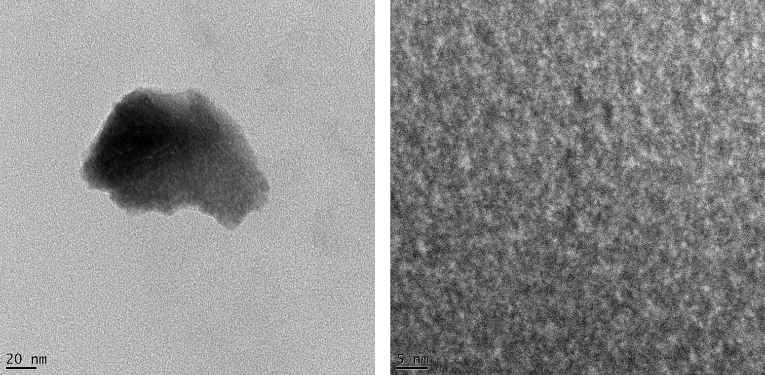


**Figure S10.** TEM images of carbon networks without phytic acid

.


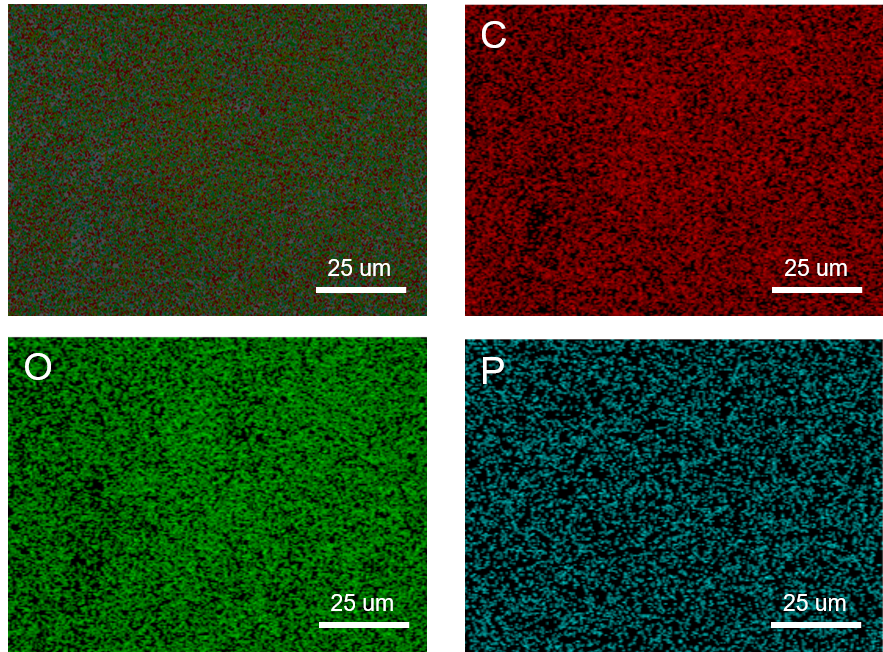


**Figure S11.** Elemental distribution in hot-pressed cellulose films.


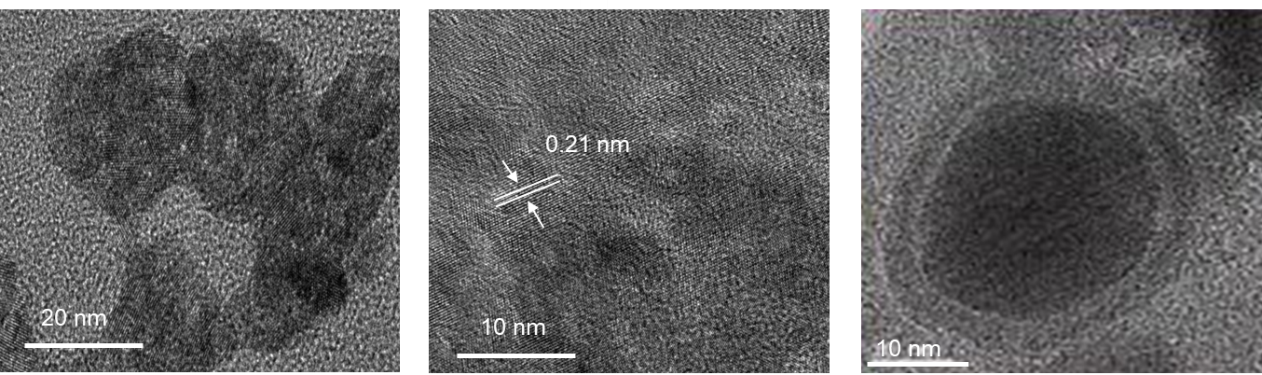


**Figure S12.** TEM of NCM carbonized from CF by employing a one-step hot-pressing method.

**Figure S13.** Raman spectra of the front and back films before and after hot pressing.


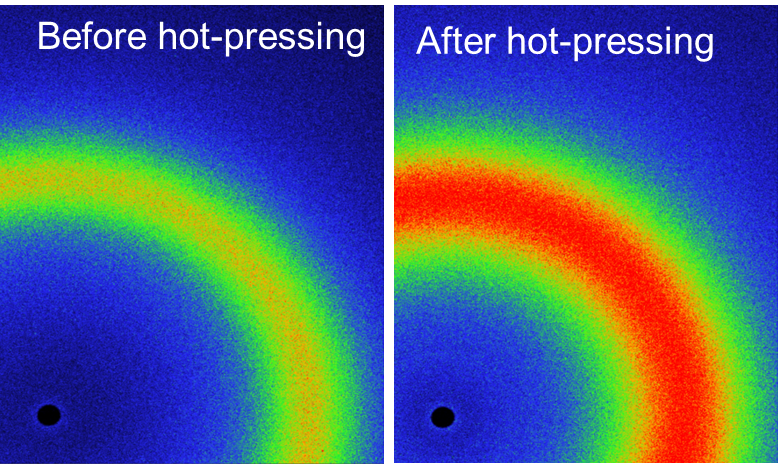


**Figure S14.** 2D-SAXS patterns of cellulose-based film before and after hot pressing.

**Figure S15.** Small-angle X-ray scattering spectra of the film before and after hot pressing.


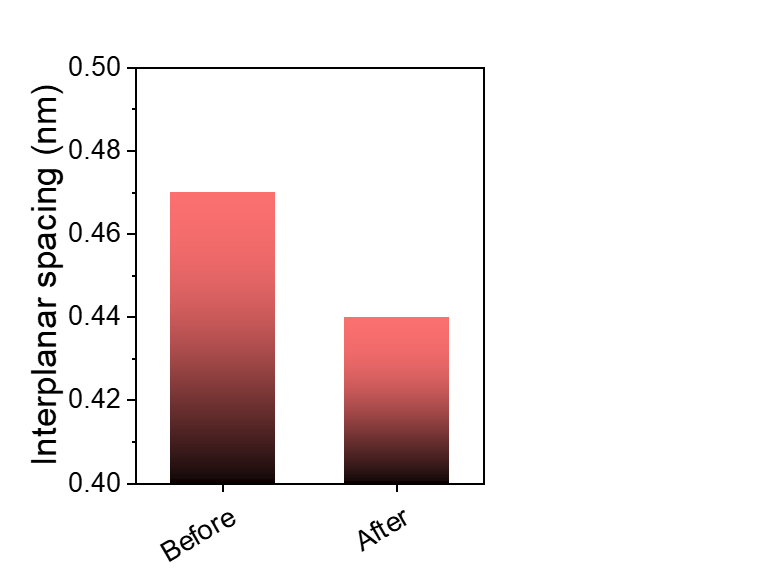


**Figure S16.** The interlayer spacing of cellulose-based film (CF) before and after hot-pressing.


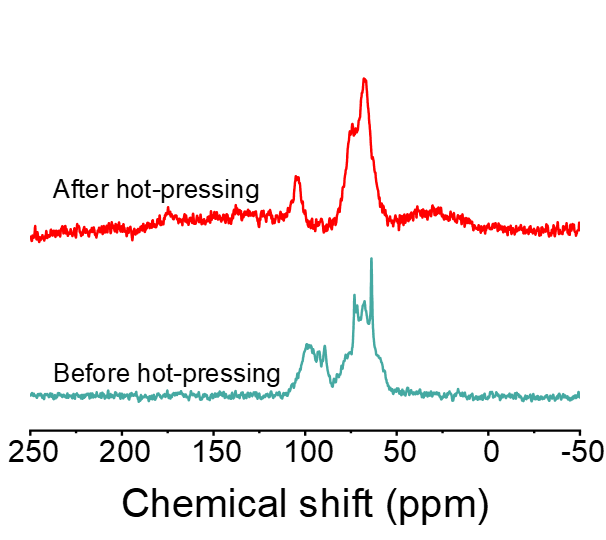


**Figure S17.** Changes in solid-state ^13^C-NMR phosphorus spectra of before and after hot-pressed films.


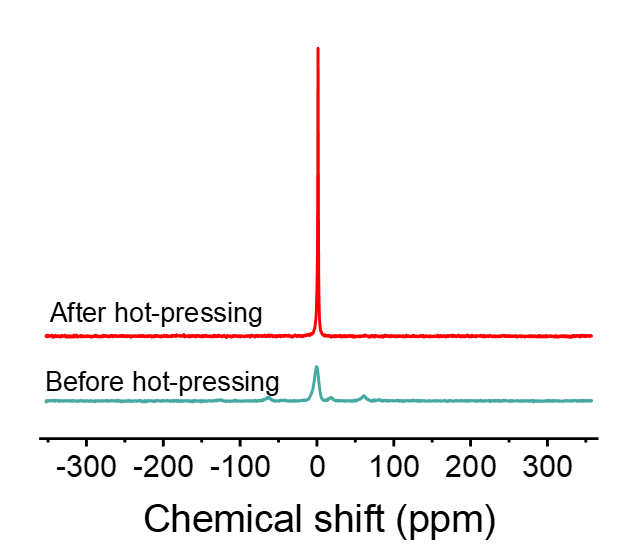


**Figure S18.** Changes in solid-state 31P-NMR phosphorus spectra of before and after hot-pressed films.


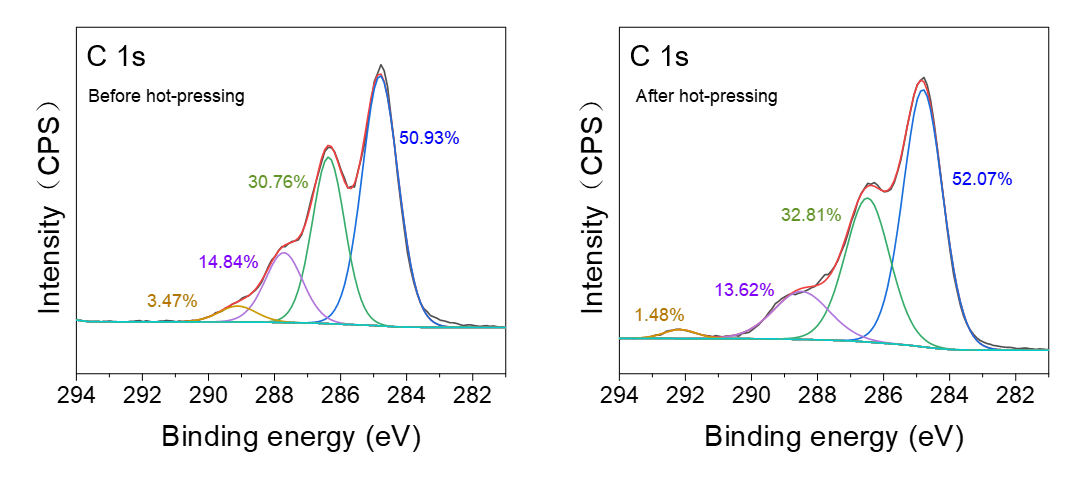


**Figure S19.** C1s energy spectra before (a) and after (b) hot pressing.


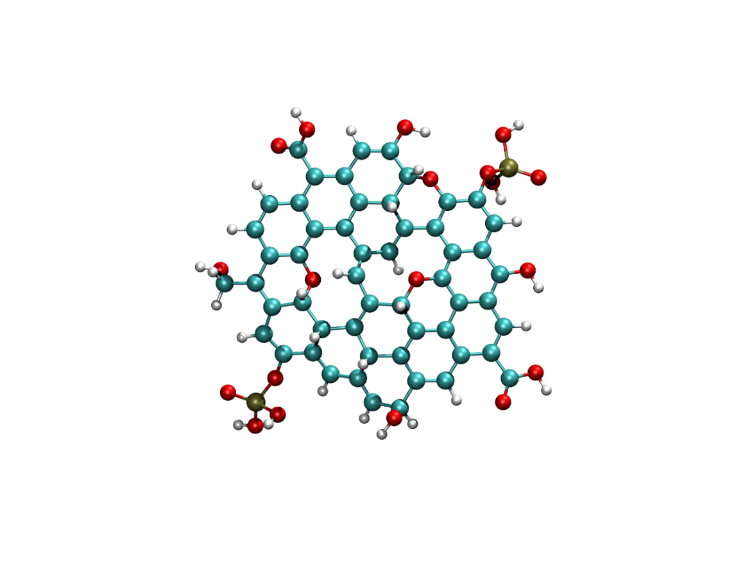


**Figure S20.** Optimized structure of a single CPDs molecule. Indigo represents carbon atoms, red represents oxygen atoms, tan represents phosphorus atoms, and white represents hydrogen atoms.


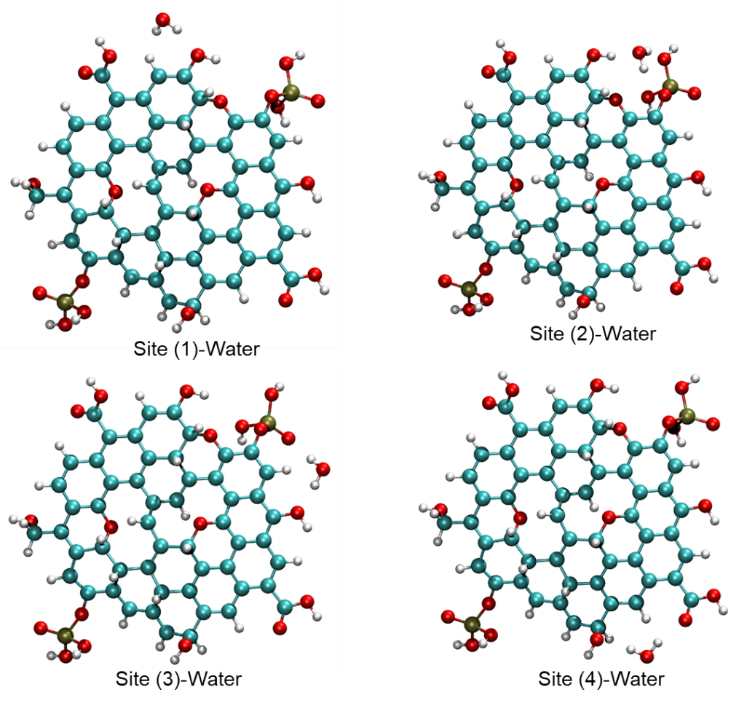


**Figure S21.** Optimized structure of a single CPDs molecule. Indigo represents carbon atoms, red represents oxygen atoms, tan represents phosphorus atoms, and white represents hydrogen atoms.


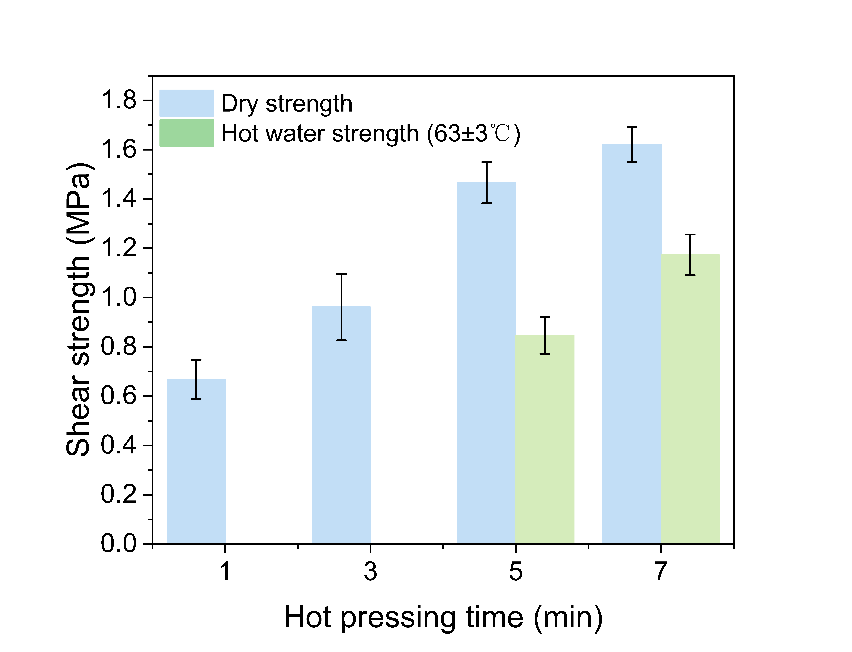


**Figure S22.** Graph showing changes in bonding strength over hot-pressing time within 1–7 minutes

.


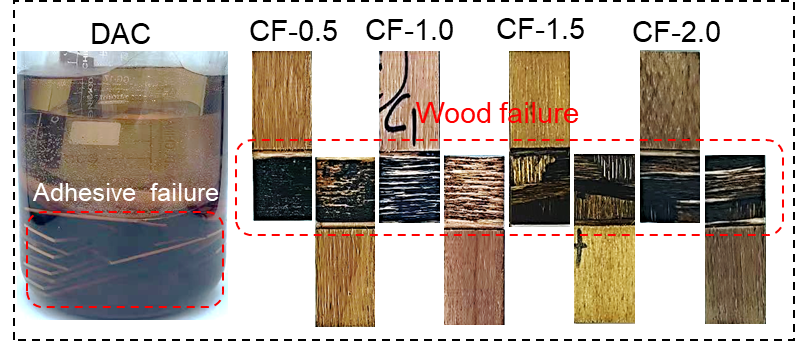


**Figure S23.** Wood-broken display of tensile specimens.


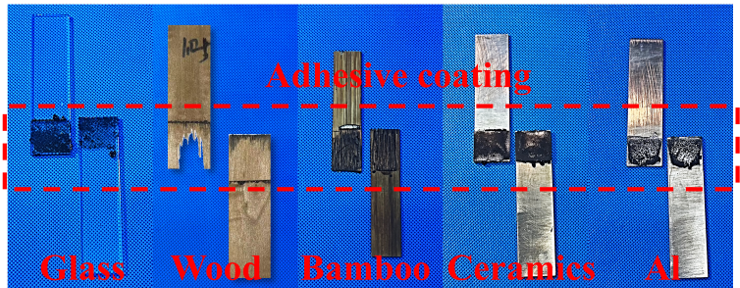


**Figure S24.** Substrate-broken display of tensile specimens.


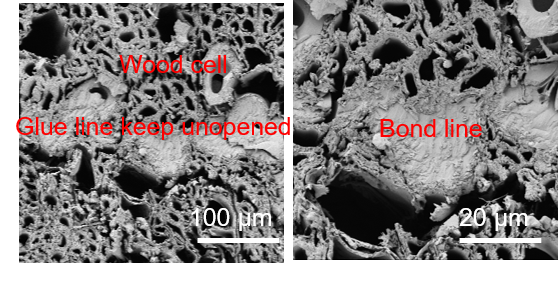


**Figure S25.** SEM image of cross-section of NCM-plywood.


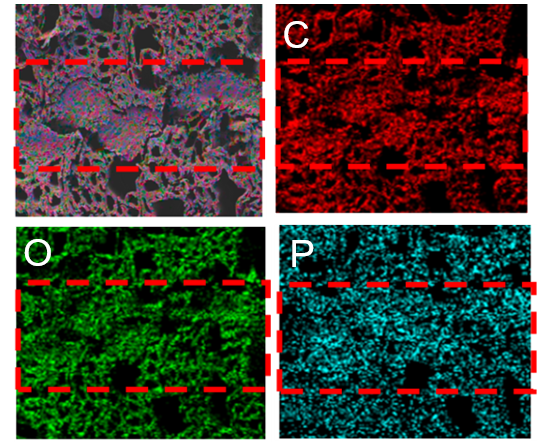


**Figure S26.** EDS image of cross-section of NCM-plywood.


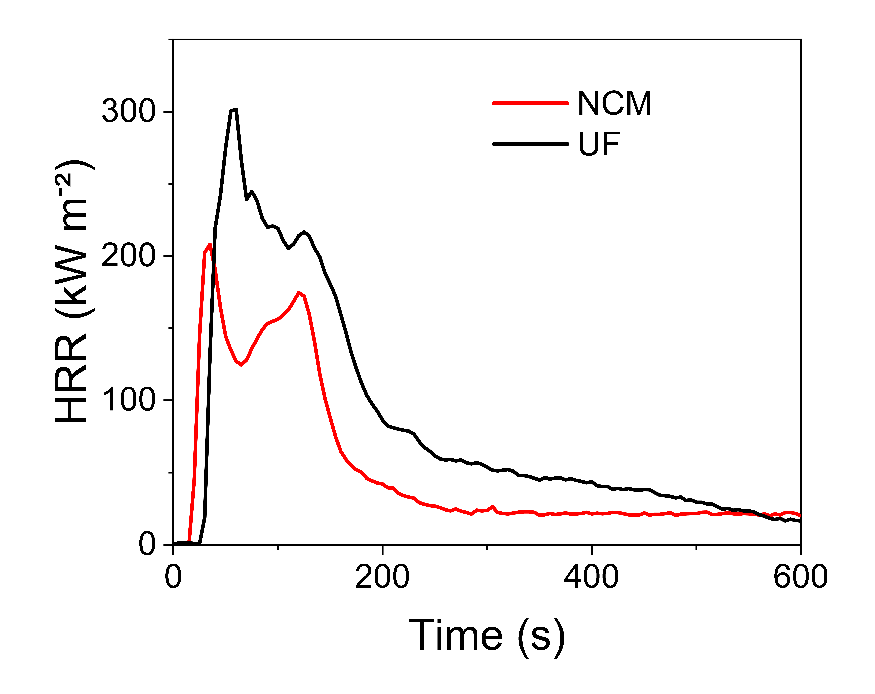


**Figure S27.** Comparison of HRR in urea-formaldehyde and membrane-prepared plywood using a cone calorimeter.

**
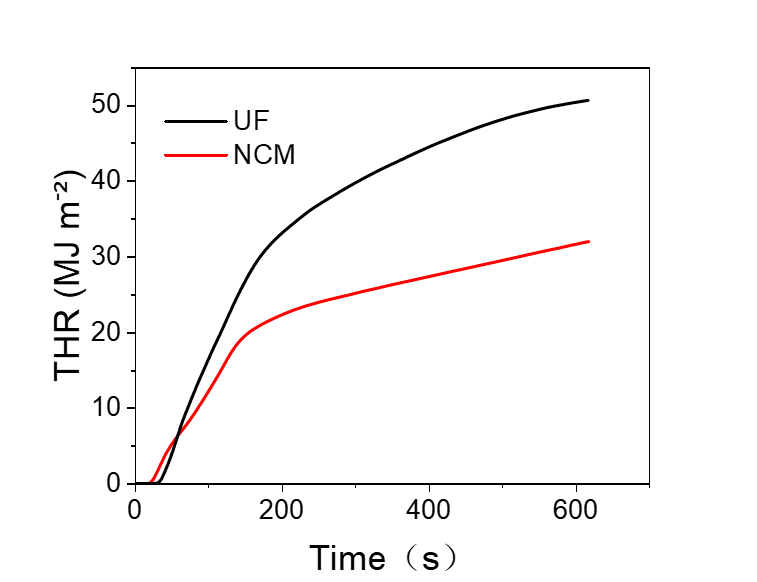
**

**Figure S28.** Comparison of THR in urea-formaldehyde and membrane-prepared plywood using a cone calorimeter.


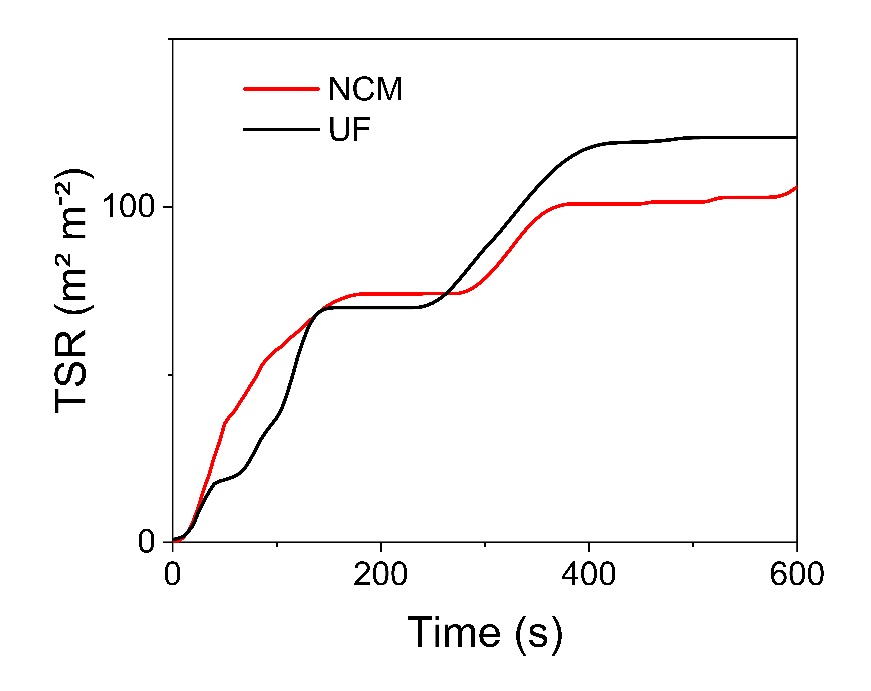


**Figure S29.** Comparison of TSR in urea-formaldehyde and membrane-prepared plywood using a cone calorimeter.

**
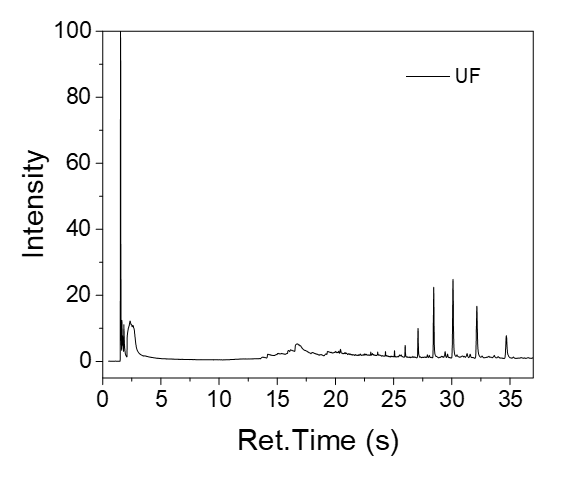
**

**Figure S30**. GC-MS Chromatogram of VOC emissions from UF Adhesive


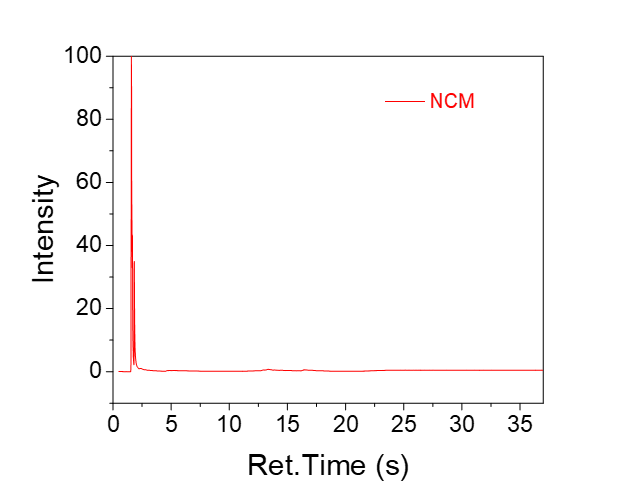


**Figure S31**. GC-MS Chromatogram of VOC emissions from NCM Adhesive

# S3. Tables

**Table S1** The formula of cellulose-based film.

| Samples | DAC/g | DW/g | PA | GI/g |
| --- | --- | --- | --- | --- |
| DAC | 5 | 92 | 0 | 3 |
| CF-0.5 | 5 | 91.5 | 0.5 | 3 |
| CF-1.0 | 5 | 91 | 1 | 3 |
| CF-1.5 | 5 | 90.5 | 1.5 | 3 |
| CF-2.0 | 5 | 90 | 2.0 | 3 |

**Table S2** Thermal conductivity of cellulose base film before hot pressing.

|  | 25℃ | 80℃ |
| --- | --- | --- |
| Thermal conductivity  (W m^-1^ K^-1^) | 0.082 | 0.166 |
| Specific heat capacity  (J g^-1^ K^-1^) | 0.451 | 0.362 |
| Thermal diffusivity  (mm^2^ s^-1^) | 0.46 | 0.18 |

**Table S3** Thermal conductivity of cellulose base film after hot pressing.

| Samples | 25℃ | 80℃ | 150℃ | 200℃ |
| --- | --- | --- | --- | --- |
| Thermal conductivity  (W m^-1^ K^-1^) | 0.08 | 0.088 | 0.099 | 0.113 |
| Specific heat capacity  (J g^-1^ K^-1^) | 0.498 | 0.718 | 0.891 | 0.924 |
| Thermal diffusivity  (mm^2^ s^-1^) | 0.163 | 0.12 | 0.11 | 0.12 |

**Table S4** Comparison of NCM and UF-Prepared Plywood in a Cone Calorimeter.

| Samples | UF | NCM |
| --- | --- | --- |
| THR | 50.63 MJ m^-2^ | 31.98 MJ m^-2^ |
| Mass lost | 2574.08 g m^-2^ | 2025.30 g m^-2^ |
| TSR | 70.79 m^2^ m^-2^ | 67.33 m^2^ m^-2^ |
